# Co‐Designing Medication Safety Interventions for Community Pharmacy: Bringing Together People Living With Visual Impairments and Community Pharmacists Using a Modified Nominal Group Technique Approach

**DOI:** 10.1111/hex.70904

**Published:** 2026-10-08

**Authors:** Isabel Adeyemi, Lisa Riste, Penny Lewis, Adam Pearson, Sumaira Naseem, Rebecca Morris, Darren Ashcroft, Maria Panagioti, Caroline Sanders, Sally Giles

**Affiliations:** ^1^ NIHR Greater Manchester Patient Safety Translational Research Centre University of Manchester Manchester Lancashire UK; ^2^ Greater Manchester Mental Health NHS Foundation Trust Manchester Lancashire UK; ^3^ Division of Pharmacy and Optometry, School of Health Sciences, Faculty of Biology, Medicine and Health University of Manchester, Manchester Academic Health Sciences Centre (MAHSC) Manchester Lancashire UK; ^4^ Centre for Primary Care University of Manchester Manchester Lancashire UK

**Keywords:** co‐production, patient safety, pharmacy

## Abstract

**Introduction:**

People living with visual impairment (VI), defined as a reduction in visual acuity or visual field that cannot be corrected by spectacles or contact lenses, experience health inequalities and are at increased risk of medication‐related safety incidents. This study aimed to identify challenges contributing to medication errors and to co‐design potential solutions with people living with VI and community pharmacy professionals.

**Methods:**

A modified Nominal Group Technique (mNGT) was conducted with seven adults living with VI and six community pharmacy professionals. The process comprised four stages: problem identification, solution generation, decision making and prioritisation, implementation and intervention development.

**Results:**

Participants identified twelve medication safety challenges and generated twenty‐four potential solutions spanning pharmacist training, patient and pharmacist resources, pharmacy consultations, system changes and assistive technologies. Following discussion and prioritisation, three interventions were selected for further development: (1) an accessible Patient Safety Guide (PSG) to support communication between pharmacists, patients and carers regarding safe medication use; (2) a training video for pharmacists to improve awareness and understanding of the needs of people living with VI; and (3) pharmacy system changes, such as alerts or pop‐up notifications, to improve identification of people living with VI and facilitate personalised support.

**Conclusion:**

Co‐design involving people living with VI and community pharmacy professionals successfully identified practical and acceptable interventions with potential to improve medication safety. The findings highlight the importance of communication, pharmacist awareness and system‐level support in reducing medication‐related risks for people living with VI.

**Patient or Public Contribution:**

People living with visual impairment (PwVI) were involved throughout the design, conduct, and interpretation of this study. A stakeholder consultation group comprising PwVI and a carer informed the development of the study protocol, accessibility adaptations, workshop structure, and supporting materials. PwVI participated directly in the co‐design workshops, identifying problems, generating solutions, and contributing to prioritisation of intervention ideas. Several PwVI also provided feedback on interpretation of findings and contributed to refining accessible outputs, including adaptations to the patient safety guide. Their involvement ensured that the research remained grounded in lived experience, and that the interventions developed reflected the priorities and practical realities of those directly affected.

## 1 Background

Vision impairment (VI) is defined as *‘a condition which causes a reduction in visual acuity or field of vision, and which cannot be corrected by spectacles or contact lenses'* [1]. People may be born with a VI, may develop one in childhood, early adulthood or later in life. Although the age‐standardised prevalence of blindness and VI has decreased over the past 30 years, this has been offset by a rapidly ageing population, resulting in an increasing number of mainly adults aged 60 years and over, with blindness and visual impairment globally [2]. Common causes of VI in this age group, include cataract, glaucoma, age‐related macular degeneration (AMD), diabetic retinopathy, and presbyopia, all of which occur more frequently after the age of 60 [3].

In 2020, 1.1 billion people were described as living with sight loss globally, 295 million having moderate to severe visual impairment (crude prevalence 3.7%), and 43 million categorised as being blind (crude prevalence 0.5%) [2]. These new data represent a rise in prevalence rates of 3% (285 million) for moderate to severe VI, and 10% rise (39 million) in those reported as blind since 2010 [4]. The largest group of people with VI (PwVI) are adults aged over 50, who represent 82% of the blind and sight impaired population worldwide [4]. In the United Kingdom, approximately 2.28 million adults (3%) were classified as having moderate‐severe sight loss, while around 171 thousand were recorded as being blind. The Royal National Institute of Blind people (RNIB) predict that the number with VI will rise to 4 million by 2050 [5]. People living with visual impairment (PwVI) are often marginalised, suffer poverty and find it difficult to access employment [6]. The global burden on lost productivity has been estimated at US $411 billion [7].

PwVI have higher health inequities and are at increased risk for medication safety errors in comparison to the general population [8] and compared with those without VI [9]. Reasons for this include difficulties in differentiating between medications, for example, different eye drop bottles, or various types of tablets/capsules, which might lead to them taking the wrong medication [10]. In addition, some older PwVI report being unable to open medication containers and forgetting to take medication on time which impact on compliance [11]. PwVI are more at risk of social isolation [12], which further exacerbates their increased risk of patient safety incidents, especially where no‐one else is present to help clarify medications. The risk for this increases with polypharmacy, that is, the more different types of medication a person takes. Across the board, PwVI report receiving sub‐optimal healthcare, such as struggling to make and access appointments, and facing barriers around accessing healthcare information, particularly around medication [13]. People who were registered blind engaged in research specifically designed to develop a measure to capture their experiences with healthcare professionals in a bid to capture some of these issues [14].

In 2016, NHS England introduced the Accessible Information Standard (AIS), which required that the information and communication needs of disabled patients are proactively addressed in all aspects of NHS healthcare, including the dispensing of medication [15]. The AIS recommend a 5‐stage approach to supporting users based on: (i) identification of needs, (ii) recording of needs, (iii) flagging of needs, (iv) sharing of needs, and (v) meeting of needs. Whilst AIS posters are often displayed in community pharmacies, the visual message that staff can help provide information in alternative sources such as braille or large font often fails to reach PwVI, so staff remain unaware of additional needs. There is little guidance on how healthcare professionals can help meet peoples' needs with only 35% of organisations reporting they ran regular AIS training, and 37% never running it [16].

In a study of 271 ‘blind people' in the USA who were asked questions about participating in Precision Medicine Research, 15% reported 1–2 barriers (24.7%) and 65% identified 3–6 barriers [17]. Underrepresentation of PwVI in research leads to a lack of data from them, negatively affecting the development of services and interventions [13]. Similar to ‘Nothing about me, Without me’ a phrase made popular after UK Health Secretary Andrew Lansleys' report on patient‐centred care in the NHS [18], the phrase was reported to have been first used by disability rights activists in South Africa as ‘Nothing about us, without us’ in 1990s and subsequently adopted by many groups who rightly demand inclusion and involvement in healthcare system change [19].

## 2 Aims

Our research not only sought to understand potential medication safety experiences among this group, but to actively involve PwVI in co‐designing solutions along with community pharmacy professionals using the Nominal Group Technique (NGT) in an online setting [20]. Bringing together pharmacists and PwVI is a novel alternative to PwVI‐only focus groups, which enables both parties to understand each other's experiences of medication harm and to co‐design solutions together which both parties are invested in. Here, we present details of the consensus methodologies used to facilitate full engagement and how we produced a patient and pharmacist consensus on feasible intervention/s that could be prioritised to improve medication safety by reducing potential future errors for PwVI.

## 3 Methods

### 3.1 Stakeholder Consultation

In March 2020 (prior to the United Kingdoms' COVID‐19 first lockdown), two of the authors (L.R. and S.G.) attended the ‘Tea and Tech’ event at Henshaws (a North‐West England sight loss charity) to discuss creating a medication safety app for PwVI. Attendees expressed their difficulties around using personal technology with failing (or in some cases zero) sight and shared that expecting them to have an ‘app’ downloaded added to their already large workload as ‘patients’ in trying to manage various health conditions. We subsequently convened a stakeholder consultation group comprising five PwVI, a carer, two medication safety researchers (S.G., R.M.) and a Pharmacist (P.L.) to return to a place of understanding people's experiences and allowing them to co‐design solutions to help overcome these.

The challenges of setting up this group during the COVID‐19 pandemic are described elsewhere, including adaptations made to facilitate participation of PwVI in remote Public and Public Involvement and Engagement (PPIE) work [6]. The group met online four times from May 2020, during which they developed the study protocol, advised on study design, including the need for participant diversity, participant information sheets, ethical aspects, and data analysis, resulting in an ethics application to conduct two co‐design workshops described below.

### 3.2 Setting

The study received ethical approval from Manchester University Research Ethics Committee on 31 March 2021. Two co‐design events, each lasting 2 h, brought together PwVI and community pharmacists (CP) with medication safety researchers, online using Microsoft Teams. Both workshops were held online during the COVID‐19 pandemic (June and July 2021), to facilitate research without the need for face‐to‐face meetings and travel.

### 3.3 Workshop Participants

PwVI were recruited from both our stakeholder consultation group, via AP personal contacts and through Royal National Institute of Blind People (RNIB) Northwest Community Connect scheme. A purposive sample ensured we captured a diverse range of PwVI, in terms of age, gender, ethnicity, type and duration of VI.

CPs were recruited from the Greater Manchester Patient Safety Community Pharmacy Patient Safety Research Collaborative. ‘The Patient Safety Collaborative’ was formed in 2015 and comprised seven CP members based in Greater Manchester, all with an interest in patient safety. Members accessed theoretical learning around patient safety models at monthly meetings, with pharmacists applying new learning in their respective pharmacies, evaluating improvements in medication safety and feeding back to the group. Participants were invited to attend both workshops, ensuring each outcome incorporated the perspective of all stakeholders.

PwVI received reimbursement of £50 as a shopping voucher per 2‐h co‐design workshop. This was in line with INVOLVE guidance at the time, now hosted by the National Institute for Health Research (NIHR) [21] and was the preferred option from participants, some of whom were in receipt of benefits. Community pharmacists received £25 per hour, which covered replacement locum costs during co‐design workshops, with these taking place during scheduled meetings of the Community Pharmacy Patient Safety Collaborative, to ensure availability. This was paid to their employing pharmacy as workshops were conducted within their normal working hours.

### 3.4 Study Design

To identify medication safety challenges and co‐design potential solutions, we used a modified Nominal Group Technique (mNGT), a structured consensus method that combines qualitative discussion with prioritisation and ranking of ideas to support decision making [22, 23]. NGT has been widely used in healthcare research and has been successfully adapted for patient safety and intervention co‐design studies involving multiple stakeholder groups [20, 23, 24].

The mNGT approach was developed collaboratively with our stakeholder consultation group and adapted to support participation by people living with visual impairment (PwVI) and remote participation during the COVID‐19 pandemic. Adaptations included accessible study materials, pre‐workshop technology support, verbal rather than visual facilitation techniques, small‐group discussions using breakout rooms, and additional facilitators and note‐takers to promote inclusive participation. These adaptations were informed by previous work involving PwVI and recommendations for conducting NGT in virtual settings [6, 25]. Table 1 summarises the adaptations made to facilitate participation by PwVI and the online delivery of the workshops.

**Table 1:** Adaptations to NGT methodology to facilitate PwVI participation and online modality.

NGT Stage	Adaptations for PwVI	Online adaptations
Planning	RNIB Vision Impairment Awareness Training (VIAT) delivered to all non‐VI participants and team	VIAT training included online etiquette for facilitators
	Stakeholder consultation group advised two co‐design workshops	Decision which platform to use for workshops
	Identification of suitable facilitators, note takers and assigning roles	Identification of suitable facilitators, note takers and assigning roles
Recruitment	Aim to have balance numbers of PwVI and CPs	
	Recruitment target 6–9 participants from each group	
	Diverse experiences, age, gender and eye conditions, duration of VI	
Piloting	Researcher phones PwVI to check technology use, see if any additional support will be required for workshop participation	Researcher calls to check technology use, see if any support required for workshops
	Check access to study documentation is large font/audio required?	Takes phone number to use on the day if there are issues getting online
	Help complete study paperwork including audio consent for participation	
Co‐design Workshops:	Introductions and establishing ground rules	Introductions and establishing ground rules
	Facilitators to monitor and ensure all participants have an equal voice	Technical support—see below
Whole Group	Use of note taker to capture ideas generated and to feedback to whole group	Camera use—see below
Co‐design Workshops:	Use of breakout rooms to enable smaller group discussions which are easier for PwVI	Use of breakout rooms to enable smaller group discussions
Small Groups	Use of note taker to capture ideas generated and to feedback to whole group	
Data analysis	Ranking of ideas on 1:1 basis in breakout room, one room for PwVI and one room for CPs.	Ranking choices entered into excel spreadsheet online only accessible to two facilitators involved in ranking
	Participants provided their ranking choices naïve to others votes	
	Facilitators fed back results of ranking to whole group—for PwVI, CP and combined	
Post event	Researcher calls PwVI to ensure were happy with participation and answer any questions and arranges reimbursement for participants	Invitation to collaborate in solution implementation offered via email
	Invitation to collaborate in solution implementation offered via email	

Traditional NGT comprises three broad stages: problem identification, solution generation and decision making, involving the generation, discussion and prioritisation of ideas [22, 26]. Our modified approach comprised four stages: (1) problem identification, (2) solution generation, (3) decision making, and (4) prioritisation, implementation and intervention development. The fourth stage extended the traditional NGT process by explicitly considering intervention feasibility, implementation and future development pathways [23].

During the decision‐making phase, we incorporated a ‘critical friend’ approach adapted from Tyler et al. [23]. A critical friend approach involves constructive challenge and scrutiny of ideas by individuals who were not directly involved in generating them, with the aim of refining assumptions, improving feasibility and strengthening proposed solutions. Owing to the relatively small number of participants within each breakout room, all participants acted collectively as critical friends during the whole‐group discussion rather than moving individuals between groups.

An approximately equal balance of community pharmacy professionals (CPs) and PwVI was sought to ensure discussions reflected both professional and lived‐experience perspectives and to minimise the risk of one stakeholder group dominating discussions.

Prior to the workshops, PwVI were contacted to identify any additional support requirements, including assistance with accessing videoconferencing software, alternative document formats and completion of study documentation. These preparatory discussions informed the decision to increase facilitator support during the workshops. PwVI were given the option of participating with cameras switched on or off, while sighted participants were encouraged to use cameras to facilitate communication. Sighted participants were also encouraged to use contrasting virtual backgrounds to support participant recognition and communication for those with residual vision. Facilitators used structured turn‐taking, verbal cues and regular verbal summaries throughout to ensure accessibility and active participation by PwVI [6].

The principal investigator (SG) chaired both workshops, supported by facilitators (L.R., P.L. and R.M.), note‐takers (M.L., A.R. and A.W.) and an additional facilitator (I.A.) who provided technical support where required. Two online co‐design workshops, each lasting approximately 2 h, were conducted 3 weeks apart using Microsoft Teams.

In Workshop 1, participants were allocated to mixed breakout groups comprising both PwVI and CPs. During Stage 1, participants identified challenges associated with medication use and medication safety. During Stage 2, groups generated potential solutions to address the issues identified. Each breakout group included a facilitator and note‐taker, with discussions captured through detailed field notes. During Stage 3, proposed solutions were reviewed, refined and combined during a whole‐group discussion using the critical friend approach [23].

Workshop 2 focused on Stage 4 of the process. Participants reviewed the solutions generated during Workshop 1, considered their feasibility and potential impact, and prioritised interventions for future development. Researchers provided a summary of Workshop 1 findings and facilitated discussion regarding implementation considerations and possible development pathways. This final stage enabled participants not only to prioritise intervention ideas but also to consider how they could be operationalised within community pharmacy practice.

### 3.5 Data Sources

Data were collected through facilitator and note‐taker records from breakout room discussions and audio recordings of whole‐group discussions. Audio recordings were used to verify and supplement contemporaneous notes and to ensure accurate capture of the issues and solutions discussed.

During the prioritisation phase, participants independently ranked the proposed intervention ideas. Rankings were collected individually by researchers to minimise the influence of group discussion on voting decisions. One researcher (I.A.) collected rankings from PwVI participants, and one researcher (L.R.) collected rankings from community pharmacy professionals. Scores were summed separately for each stakeholder group and subsequently combined to derive overall rankings. Consistent with NGT methodology, these rankings were used to guide discussion regarding intervention feasibility and priorities for future development rather than as a standalone measure of importance [22, 23].

## 4 Results

### 4.1 Participants

Six CPs attended both events. Seven PwVI attended the first event, and six out of the initial seven attended the second event. Table 2 lists the demographics of PwVI. Six participants with VI were female, one was male. Six participants were of White British ethnicity, one reported being from another non‐specified white ethnicity. Data on VI condition and age is missing for one participant. The mean reported age of participants with VI was 40.2 and ranged from 23 to 58 (Table 2).

**Table 2:** Demographics for participants with VI.

Participant number	Type of VI condition	Details on loss of vision	Year diagnosis was given or whether congenital
1	Retinitis pigmentosa	The participant has peripheral loss in their vision. This can be described as tunnel vision	congenital
2	Leber congenital amaurosis	This genetic condition reduces acuity (sharpness) of vision and can result in loss of colour vision.	congenital
3	Diabetic retinopathy and ulcerative keratitis	There are black spots on field of vision.	2018
4	Alström syndrome	Alström syndrome is photophobia and retinal degeneration, this means that both field of vision and acuity are affected. This participant reported total blindness in one eye severe sight impaired in the other eye.	congenital
5	Stargardt disease	Stargardt disease affects central field of vision	2008
6	Optic discs and optic nerves in both eyes damaged.	Large swathes of fields of vision appear black. Vision was darker.	No diagnosis, symptoms began 2019

### 4.2 Workshop 1

The chair introduced the project, summarised what was already known about PwVI trying to manage their medications (drawing from the authors' scoping review) [27], and previous stakeholder involvement work. Whilst this could have been achieved as a whole group activity, participants were allocated to one of three small discussion groups using break out rooms, to facilitate PwVSI sharing their own personal experiences, expressing their views and suggesting solutions with an equal number of CPs to PwVI.

#### 4.2.1 Phase 1: Problem Identification

The number of groups identifying each issue is presented to demonstrate convergence across independently facilitated discussion groups and should not be interpreted as a measure of the relative importance of the issues identified (Table 3).

**Table 3:** Problems identified by small groups and the numbers of groups that identified each issue.

Problem	Number groups identifying this problem
Difficulty in identifying tablets. This includes:	3
– difficulty in memorizing the feel of tablets and the dosages – using different brands of medicines which can come in different shapes and sizes that make identifying individual tablets difficult	
Dispensing stickers get put over braille on manufactured boxes	3
– inability to access braille information	
Difficulty in identifying medication packaging‐	3
– Generic individual boxes are used for medications. There is no standard size for the boxes and no information in braille is included. – Challenges in checking if the medicine is in or out of date	
Difficulty in administering oral liquid medications preparations due to difficulties in accurately measuring required quantities.	3
Very few PwVI can read braille	2
People who have their prescriptions delivered via a courier have little or no contact with pharmacy staff. This means that there is less opportunity to communicate with pharmacists.	2
Technical solutions, such as read aloud software apps, are not effective with all types of text. Specifically:	1
– text may be too small on dispensing labels/product information (safety) leaflets – apps (e.g., Seeing AI) do not work on foil‐backed medication strips (as light from mobile phone camera bounces off).	
The pharmacist makes changes to medication brand without informing patient (changes to brand may be done due to changes in costings and or stock availability).	1
The GP makes changes to medication (e.g., dose) without informing patient	1
Difficulty with using medication blister packs. Blister packs can also be difficult to open for adults with VI and tablets may fall on the floor.	1
Difficulty in communicating	1
– Pharmacists are not aware that their PwVI uses their services. – PwVI do not mention their blindness and it may not be obvious to pharmacists. For some PwVI, they may feel uncomfortable to have to tell the pharmacist about their vision impairment.	
Difficulty in administering injections because:	1
– no means to check expiry date – no means to check whether the medication is safe to administer, such as checking whether the liquid is cloudy or not – method of administering may be difficult for PwVI, for example, to pinch self and press injection pen to administer.	

Twelve unique problems were identified across the three groups independently, six being identified between two or more groups (Table 3). The most commonly identified were:

1. **Difficulty identifying tablets**—This included difficulty remembering the feel of tablets and their dosages, a lack of continuity around the medication brand dispensed, difficulty checking medication expiry dates. When identified problems were inter‐related, they compounded medication errors. For example, medication supplied in dosette boxes (also known as blister packs, where tablets are pre‐grouped according to time of day/day of week they are to be taken) could make it harder for PwVI to identify medication errors especially where the pharmacist supplies a different brand of medication without informing the patient, or in the case where a GP makes changes to a medication dosage without informing the patient. PwVI were concerned about the safety of changes in the branding, or dosage of medication when these had not been discussed with them and had been provided in blister packs. PwVI reported that they felt blister packs necessitated that they recognise medication tablets by touch only as there are no other visual cues available from packaging.
2. **Dispensing stickers obscuring braille on medication boxes**—Two groups highlighted that very few adults can read braille. Around seven per cent of people who are registered blind or partially sighted use braille [28], and this percentage is lower still for older adults who have become more reliant on read aloud technology. Some PwVI reported being able to distinguish between tablets based on first letter of braille name.
3. **Tablets supplied as ‘split packs' (in generic white boxes) do not have braille information**—and these can also be confused with pet medication, also often supplied in this format.
4. **Difficulties measuring oral liquids dose**—where a measured spoonful was required people reported likelihood of underdosing, or spilling medicine resulting in a shorter treatment course. For those needing to use a measured syringe dose this proved challenging due to small increments meaning it was likely to be administered inaccurately.

One discussion group highlighted problems with technical solutions such as software apps to read medication labels. In reflecting on this finding, our stakeholder consultation group noted that many PwVI prefer low technological solutions for everyday use, such as adding varying numbers of elastic bands across each medication bottle to differentiate them.

#### 4.2.2 Phase 2: Solution Generation

Note‐takers provided a summary of the problems discussed in phase 1. Problems were grouped into three overarching categories: medication‐related, pharmacy‐related, and communication challenges (between CPs and PwVI). Each of the breakout groups was allocated one category area, led by the facilitator with the greatest knowledge in that area to stimulate discussion of potential solutions. Potential ideas were generated across the groups, including: training for pharmacists, technical solutions (e.g., talking labels, pharmacy system alerts), one to one consultations and support for PwVI and carers. Although solutions to the issues were generated in small groups, many of the proposed solutions were overlapped between groups, with some solutions appearing to target the same process. Table 4 shows the generated solutions and illustrates the interlinkage.

**Table 4:** Proposed solutions from Workshop 1.

	Problems		
	1. Medication‐related problems	2. Pharmacy‐related problems	3. Communication (CPs and PwVI)
**Proposed solution**	PwVI wanted more information on safer medication taking.	Raise pharmacist awareness of asking PwVI about communication support needs to identify PwVI.	Pharmacy training to encourage more pro‐active communication with PwVI. Specific training to increase empathy—video ‘in my shoes’.
	Notify PwVI when brands change.	A pharmacy resource to raise awareness of aids to improve safer use of medicines in PwVI.	Communication support for PwVI to discuss medication issues and possible solutions.
	Provide larger text font on packaging.	Offering 1:1 consultations to PwVI about adaptations/aids dependent on patient need (i.e., bigger font, colours, stickers, luminous stickers, talking labels, etc).	System changes to increase pharmacist's awareness of VI, e.g., logo, system flags.
	Go through medication with PwVI face‐to‐face (over phone if delivered).	Individual action plans for PwVI, reviewed at agreed intervals to assess the usefulness of implemented aids and prescription changes.	Make medication packaging easier for everyone, use technology, e.g., talking labels.
	PwVI being more proactive.	Identification of PwVI through the GP	
	PwVI disclose VI.	Standard recording of this information in the patient records with subsequent alerts on future dispensing for the patient.	
	PwVI ask for support around using medication.	Medication suppliers—Raising issue regarding VI friendly packaging with major suppliers	
	PwVI remembering names of medication.		
	Using summary care records to identify PwVI.		
	Pop up notifications of PwVI		
	Record individual needs		
	Braille/talking stickers/labels		

#### 4.2.3 Phase 3: Decision Making

Facilitators together with note‐takers provided a summary of the potential solutions discussed in the smaller breakout room groups. Adopting a modification of the ‘critical friend approach’, where one person would normally transfer groups to help refine thinking, our potential solutions were discussed with the whole group. As some of the solutions generated were interlinked as shown in Table 4, some ideas were combined.

Before the second workshop, the stakeholder consultation group reviewed the solutions that had been generated. They thought the solutions generated were sufficient and did not have any of their own to add.

### 4.3 Workshop Two

Through discussion about feasible interventions that could be taken forward, seven unique ideas for interventions were proposed for ranking, and the results are shown in Table 5.

**Table 5:** Voting by CP and PwVI on the seven proposed solutions from the co‐design workshops.

Proposed solutions from the co‐design events	Total cumulative score for CPs	Total cumulative score for PwVI	Total combined score CP + PwVI
Offering one to one consultations to PwVI about possible aids to assist safe medication use dependent on the specific preferences and needs to the patient (i.e., bigger font, braille, colours, luminous stickers, talking stickers/labels, etc.)	21	29	50
Make medication packaging easier for everyone, use technology, e.g., talking labels	19	29	48
Production of a training video for pharmacists to increase empathy towards PwVI, e.g.,—video ‘in my shoes’	23	23	46
System changes to increase pharmacist's awareness of VI, e.g., logo, system flags, pop up notifications	22	18	40
The use of individual action plans for PwVI, reviewed at agreed intervals to assess the usefulness of implemented aids and prescription changes	20	20	40
Pharmacists to go through medication with PwVI face‐to‐face (over the phone if delivered).	24	14	38
Raising issues about friendly packaging with major suppliers	11	7	18

#### 4.3.1 Phase 4: Prioritisation, Implementation and Intervention Development

Each participant ranked each proposed solution, with a rank of 7 being their most favoured one to take forward and 1 being their least favoured. Table 5 shows the seven proposed intervention ideas, the cumulative scores allocated to each from pharmacists and from PwVI, and the total score.

The three highest ranking solutions when combining CP and PwVI rankings were: (1) offering one to one consultations to PwVI about possible aids to assist safe medication use dependent on the specific preferences and needs of the patient, (2) making medication packaging easier for everyone through the use of technology, for example, talking labels, (3) production of a training video for pharmacists to increase empathy towards PwVI.

After identifying the three highest‐ranked solutions, participants discussed their feasibility and implementation. Although technology‐enabled packaging solutions ranked highly, participants considered them difficult to implement because they relied on manufacturer engagement, additional infrastructure and specialist equipment. In contrast, pharmacy identification systems (e.g., alerts, flags and pop‐up notifications) were viewed as a feasible and actionable mechanism for identifying PwVI and enabling personalised consultations. As a result, this intervention was prioritised for further development alongside the Patient Safety Guide (PSG) and pharmacist training video.

Below is a summary of the discussion of how CPs and/or PwVI could implement these three interventions in practice.

1. **Offering one‐to‐one consultations to PwVI**
  In order for one‐to‐one consultations to take place, CPs had to be able to identify who had a VI. CPs and PwVI highlighted communication challenges as a key underpinning this with their being an expectation that CPs who had access to medications lists would also have access to medical history so be aware of their VI. This misconception, along with reluctance to talk in public about VI, which increased their feelings of vulnerability and potential distress, was a mismatch compared with CP expectations that PwVI would inform them if they needed support. Both PwVI and CPs recognised that CPs did not have time to ask everyone using the pharmacy if they needed additional support or not.
  One‐to‐one consultations had to be tailored to the needs of the PwVI, and that PwVI could be given tools to support this discussion as well. This linked with existing work to develop and pilot existing patient safety guide for patients and carers to support patients and carer to identify key points where they can make their care safer and address key patient safety questions whilst adopting a broader view of patient safety to support effective communication [29, 30].
  In discussion with PwVI, it was agreed to adapt the existing guide to ensure its accessibility, with PwVI reporting that the guide would be useful to support autonomy in their interactions with pharmacists and other primary care providers and their involvement in their care and safety. Additional patient and public involvement work was supported by the NIHR GM PSTRC to adapt the guide (e.g., making an extra‐large, easier to read format version and suggestions about aids to support PwVI to assist medication safety).
2. **Producing an** ‘***in your shoes***’ **training video for pharmacists.**
  Discussions from the co‐design group, along with additional stakeholder group consultation, allowed us to develop a training video script. This focused on empathy and understanding that encompassed some of the difficulties reported by PwVI that they felt needed to be shared with their pharmacist. We subsequently contracted The University of Manchester Medica Centre to film a scenario in the pharmacy belonging to one of our community pharmacists who had taken part in the co‐design workshops. An actor with visual impairments portrayed the role of a PwVI in the video, alongside one of the community pharmacists. One PwVI who had participated in the co‐design workshops agreed to be filmed discussing her use of the Patient Safety Guide (PSG).
3. **System changes to increase pharmacist's awareness of VI, for example, logo, system flags, pop up notifications**

To be able to deliver the most highly ranked solution, 1:1 consultation, pharmacists highlighted their need to have a mechanism to identify PwVI attending the pharmacy, and that they were not party to this information. Discussions focused on whether a pharmacy pop‐up alert that utilised the shaded eye logo belonging to The Partially Sighted Society (that had been seen during the scoping review undertaken for this work) could help identify who should be offered a consultation in community pharmacy. Funding was subsequently awarded to scope this work, by The University of Manchester, Masood Entrepreneurial Centre via a Starter Award. In response to concerns from both pharmacists and PwVI it was agreed to also develop a website to act as a repository of helpful strategies and resources, aligning with priority 3 around creating pharmacy training resources.

The initial stakeholder consultation group went on to help oversee the development of the interventions following the conclusion of workshop two.

## 5 Discussion

### 5.1 Summary of Findings

This study used a modified NGT and co‐design approach to identify solutions for medication errors in PwVI. Two NGT events were held with pharmacists and PwVI to develop ideas on how to reduce medication errors PwVI. Participants identified twelve unique problems/challenges that could lead to medication errors, they proposed twenty‐four unique solutions that targeted training pharmacists or providing resources for pharmacists and PwVI, consultations and system changes in pharmacies, or technical solutions. From these proposed solutions, seven intervention ideas were put forward for ranking. Ranking guided discussion on what interventions had the most promise for effectiveness and feasibility and informed decision making for which three solutions to take forward for intervention design: 1. To develop an accessible patient safety guide for patients and carers to address communication challenges between pharmacists and PwVI in discussing safe medication use tailored to the individual needs to the patient, and to support PwVI's autonomy in their interactions with pharmacists., 2. Producing a training video for pharmacists. 3. system changes to increase pharmacists' awareness of VI, such as pop‐up notifications to identify PwVI.

We achieved excellent ongoing collaboration following these events, with two PwVI volunteering to help drive forward each of the three suggested solutions. Feedback from them was that their involvement in this work helped them to feel useful both to the NHS, and the wider VI community. Two CPs assisted with our post‐event work, one offering his pharmacy and agreeing to be filmed, and another with an interest in pharmacy software systems agreeing to join the team developing a solution to identify PwVI in community pharmacy settings. Two public contributors continued their involvement to support the adaptation of the patient safety guide for PwVI. These collaborations have helped secure additional funding outside that funded by the NIHR GM PSTRC for ongoing work.

### 5.2 Previous Literature

Previous research has highlighted the importance of assistive products, accessible medication information, and adherence supports to help people living with visual impairment (PwVI) manage their medicines safely [11]. More recently, Delphi consensus studies have proposed broader system‐level recommendations to improve medication safety and inclusive pharmacy care for PwVI, including pharmacy workforce training, communication adaptations, identification and recording of accessibility needs, use of assistive technologies, and organisational changes within pharmacy services to better support accessibility and inclusion [31, 32].

Our findings are consistent with these recommendations and extend them through a co‐design process that brought together PwVI and community pharmacy professionals to jointly identify, refine and prioritise interventions. While previous consensus studies have identified what pharmacy services should provide to improve accessibility and medication safety, our study adds insight into which interventions stakeholders consider most acceptable, feasible and relevant within routine community pharmacy practice. In particular, participants prioritised pharmacist training, enhanced communication, personalised support through consultations, and systems to identify PwVI within pharmacy workflows.

The prominence of pharmacist training, improved communication, and system‐based identification mechanisms in our findings reinforces emerging evidence that medication safety for PwVI extends beyond assistive technologies alone and requires changes at individual, organisational and service levels [31, 32]. Participants viewed community pharmacists as an important and accessible source of support but identified communication barriers and difficulties identifying people who may require reasonable adjustments as key obstacles to safer medication use. The prioritised interventions, therefore, focused not only on supporting medicine‐taking behaviours but also on improving interactions between pharmacy staff and PwVI and enabling services to respond more effectively to individual needs.

This study demonstrates that a modified NGT and co‐design approach can be successfully used to identify and prioritise interventions to improve medication safety for PwVI. Bringing together community pharmacists and PwVI provided opportunities for shared learning, enabling participants to develop solutions that were informed by both lived experience and practical knowledge of pharmacy systems. Within the context of the COVID‐19 pandemic, the online format also demonstrated the feasibility of conducting accessible co‐design activities remotely, while generating intervention ideas that subsequently informed further development work.

### 5.3 Strengths and Limitations

#### 5.3.1 Strengths

A strength of this work is that it brought together the opinions of PwVI and CPs. In particular, one strength was in recruiting PwVI with a range of visibility/sight loss, such as peripheral vision, central vision, and total blindness. Having adults with different types of VI was helpful in providing their different experiences (and had been cited as a strength in the design stage of the study by our Stakeholder consultation group). Having an equal number of PwVI and CPs was a strength, given it helped ensure that neither side dominated the other during discussions.

At the time of data collection, with so much research stalling in the face of travel restrictions and face‐to‐face meetings being limited by Covid‐19, we were able to use online meeting facilities in order to help identify and suggest areas of further work to help improve medication safety for PwVI. The adaptations made for working with PwVI were another strength, which ensured everyone's voice was heard in a way that is appropriate to this patient group. This was also from input of the Stakeholder consultation group, who helped to design the study and the NGT events.

### 5.4 Limitations

A limitation of the research study was that there was no option for participants to have face‐to‐face contact for data collection. To date, there is no clear consensus as to whether face‐to‐face or online focus groups have greater advantages for the data produced [33]; however, in the current study participant input in the virtual setting helped ensure all voices were heard during workshops [6]. Nonetheless, the use of a technology could have been a barrier for some PwVI to participate, particularly those who were digitally excluded. Those selected by virtue of their relationship with RNIB had been trained by them to use Microsoft Teams during their often long‐standing relationship. PwVI who are less familiar with technology, and in particular older adults, may have been less likely to participate. In our study, the oldest participant with VI was aged 58; however, data show 79% of PwVI are aged above 65 [5]. Consequently, the views of older adults living with visual impairment and those experiencing digital exclusion may be underrepresented in our findings. The participant group also lacked demographic diversity, comprising predominantly female participants and individuals of White ethnicity. Consequently, the findings may not capture the full range of experiences and support needs of people living with visual impairment from other demographic backgrounds.

Despite participant numbers in the study being small, this was a necessary adaptation that enabled us to provide the appropriate support to all participants who needed it. It is unclear whether the addition of further participants to the NGT process would have generated more unique ideas to solutions. Discussion with our Stakeholder consultation group, however, suggested that the list and solution ideas were exhaustive.

The modified NGT approach adopted in this study differed from previous research [23]. In the decision‐making phase of the modified NGT, Tyler et al's [23] approach involved moving two people from each group for them to act as a ‘critical friend’ in discussing the feasibility of potential ideas. In the current study, Participants remained in their initial breakout groups to facilitate small group discussions and came together as a whole group to discuss the feasibility of potential ideas. Due to the small number of participants in each breakout group meant it would be difficult to move a ‘critical friend’ between breakout rooms, and so at the end of the workshop facilitators shared ideas from each group with all participants acting in the ‘critical friend role’, responding to the ideas generated.

In previous modified NGTs [23], participants provided their rankings anonymously. In this study, although PwVI could not provide their own anonymised ranking, one by one, they provided their responses to one of the research team (L.R.). To ensure equity, the same approach was used to elicit responses from the CPs. Although unaware of how others had ranked solutions, their responses could have been associated with social desirability bias, where participants provide responses they believe will make them look good to others, rather than revealing their true opinions or experiences [34].

Although this study focused on community pharmacy interventions, it would have been helpful to have also involved GPs or other prescribers in the workshops so that the whole of the medicines journey could have been incorporated as part of the solutions. Whilst acknowledging the inherent problems of inter‐staff communication and time pressure, from the pharmacist's perspective, it would be helpful to be asked for help where necessary, but it seems that this would be hard to achieve. Without this, the focus tends to be more upon dispensing of medication and how PwVI use their medicines at home, although this still potentially has opportunities to improve medication safety for this at‐risk patient group.

## 6 Conclusions

This study is novel in that it sought not only to identify problems leading to medication errors for PwVI but also to generate solutions/concepts that could be used to improve medication safety for PwVI by bringing them together with CPs to co‐design potential interventions.

## Author Contributions

**Isabel Adeyemi:** methodology, writing–review and editing, project administration, writing–original draft. **Lisa Riste:** writing–review and editing, formal analysis, methodology. **Penny Lewis:** writing–review and editing, methodology. **Adam Pearson:** writing–review and editing. **Sumaira Naseem:** writing–review and editing. **Rebecca Morris:** methodology, writing–review and editing, formal analysis. **Darren Ashcroft:** conceptualization, writing–review and editing, supervision, funding acquisition. **Maria Panagioti:** conceptualization, writing–review and editing, supervision, funding acquisition. **Caroline Sanders:** conceptualization, funding acquisition, supervision, writing–review and editing. **Sally Giles:** conceptualization, writing–original draft, writing–review and editing, methodology, formal analysis, project administration, supervision.

## Ethics Statement

Ethical approval was obtained from the University of Manchester Research Ethics Committee (Ref: 2021‐11098‐18381).

## Consent

Informed consent was obtained from all participants prior to their inclusion in the study.

## Conflicts of Interest

The authors declare no conflicts of interest.

## Data Availability

The authors have nothing to report.

## 1

1. NGT Approach

Phase 1: Problem Identification

Participants were asked to discuss and outline the problems associated with medication management for PwVI, focusing on adults. This comprised the dispensing of medications from pharmacies through to medication management at home.

Phase 2: Solution Generation

Participants were asked to generate as many potential ideas as possible for how pharmacists could support PwVI to manage their medication safely. In small groups, participants were asked to discuss potential solutions and the pros and cons of each proposed solution; for example, whether the proposed solution is affordable, practical, and its potential impact on medication safety. Participants were asked to generate ideas without being bound to notions of feasibility, finance, or execution.

Phase 3: Decision Making

The decision‐making phase involved participant discussions in both the small groups, and discussion as one large group. Decision making was achieved by (1) recording ideas in small groups for problems associated with medication management for PwVI and potential solutions; (2) discussing these ideas in small groups; (3) deciding in small groups on what potential solutions are feasible; (4) prioritising three to five ideas to present to the wider group; (5) reflections on feasibility of each idea as a whole group, with each group acting as a critical friend towards the ideas generated from the other group. The facilitators presented the ideas to the whole group, and their discussion was audio‐recorded with their consent.

Phase 4: Prioritisation, Implementation, and Intervention Development

There were four distinct elements to this phase: (1) idea reduction:‐ this entailed small group discussion to reduce the long list of ideas generated from the last event to a manageable and feasible list of 10 items or less. (2) individual ranking of the top ten preferences from the list (3) discussion of group rankings and generation of best three ideas and (4) discussion of how pharmacists and/or PwVI could implement these three interventions in practice. Proposed solutions were ranked individually by pharmacists and PwVI. A higher ranking indicated their most favoured one to take forward, and the lowest rank indicated their least favoured. Scores were calculated collectively to give a total score for each item across the group. The final step involved discussion on how any intervention/s might look in practice.

Table A1

**Table A1:** A comparison of NGT approaches and study adaptations for PwVI.

Stages of Nominal Group Technique [22]	Stages of modified Nominal Group Technique [23]	Activities of modified Nominal Group Technique [23]	Adaptations of activities of modified Nominal Group Technique for PwVI
Problem identification	Define	Outlining the problems	Instead of individual silent generation of ideas, participants were divided into break‐out rooms.
			Facilitators asked for contribution from each attendee.
			Note takers recorded discussion points and ideas
Solution generation	Discover	Silent generation of ideas	Ideas generated via small group discussion in break out rooms.
			Chair asked for contribution from each attendee.
			Note‐takers recorded discussion points and ideas
Decision making	Determine	Recording of ideas, discussion, decision making, prioritisation	‘Critical friend’ approach was undertaken during the whole group discussion with all participants present.
	Prioritization, implementation and intervention development	Further reducing and prioritising ideas generated in previous phase.	Ranking was not completely anonymous in that the researcher met with participants individually online to rank the proposed intervention ideas.
